# Experiences of eating disorders from the perspectives of patients, family members and health care professionals: a meta-review of qualitative evidence syntheses

**DOI:** 10.1186/s40337-021-00507-4

**Published:** 2021-12-04

**Authors:** Sanna Aila Gustafsson, Karin Stenström, Hanna Olofsson, Agneta Pettersson, Karin Wilbe Ramsay

**Affiliations:** 1 School of Law, Psychology and Social Work, Örebro University, University Health Care Research Center, 701 82 Örebro, Sweden; 2 Swedish Agency for Health Technology Assessment and Assessment of Social Services, P.O. Box 6183, 102 33 Stockholm, Sweden; 3 Sweden’s Innovation Agency Vinnova, Mäster Samuelsgatan 56, 101 58 Stockholm, Sweden

**Keywords:** Eating disorders, Anorexia nervosa, Evidence synthesis, Qualitative research, Meta-review, Meta-synthesis

## Abstract

**Background:**

Eating disorders are serious conditions that cause major suffering for patients and their families. Better knowledge about perceptions of eating disorders and their treatment, and which factors that facilitate or hinder recovery, is desired in order to develop the clinical work. We aimed to explore and synthesise experiences of eating disorders from the perspectives of those suffering from an eating disorder, their family members and health care professionals through an overarching meta-review of systematic reviews in the field.

**Methods:**

A systematic literature search was conducted in the databases PubMed, PsycInfo, Scopus, and CINAHL. Inclusion criteria were systematic reviews of qualitative research on experiences, perceptions, needs, or desires related to eating disorders from the perspective of patients, family members or health care professionals. Systematic reviews that fulfilled the inclusion criteria were assessed for relevance and methodological limitations by at least two researchers independently. The key findings were analysed and synthesised into themes.

**Results:**

We identified 17 systematic reviews that met our inclusion criteria. Of these, 13 reviews reported on the patients’ perspective, five on the family members’ perspective, and three on the health care professionals’ perspective. The study population in the reviews was predominantly girls and young women with anorexia nervosa, whilst systematic reviews focusing on other eating disorders were scarce. The findings regarding each of the three perspectives resulted in themes that could be synthesised into three overarching themes: 1) being in control or being controlled, 2) balancing physical recovery and psychological needs, and 3) trusting relationships.

**Conclusions:**

There were several similarities between the views of patients, family members and health care professionals, especially regarding the significance of building trustful therapeutic alliances that also included family members. However, the informants sometimes differed in their views, particularly on the use of the biomedical model, which was seen as helpful by health care professionals, while patients and family members felt that it failed to address their psychological distress. Acknowledging these differences is important for the understanding of anorexia nervosa and other eating disorders, and may help clinicians to broaden treatment approaches to meet the expectations of patients and family members.

**Supplementary Information:**

The online version contains supplementary material available at 10.1186/s40337-021-00507-4.

## Plain English summary

The current paper brings together existing knowledge on experiences of eating disorders. We were interested in the views of patients, family members and health care professionals. A literature search identified 17 systematic reviews which addressed these questions. The identified research focused mainly on girls and young women with anorexia nervosa, while research on other eating disorders was limited. Overall, this review suggests that it is important to acknowledge that patients, family members and health care professionals may have different experiences and views regarding treatment of eating disorders, and that it is important to consider all these views in the development of the care of eating disorders.

**Supplementary Information:**

The online version contains supplementary material available at 10.1186/s40337-021-00507-4.

## Introduction

Eating disorders (EDs) are serious psychiatric conditions that often have both psychological and physical consequences and significant societal costs [1, 2]. An ED can lead to social problems and reduced quality of life for both the victim and his or her family [3]. The debut is often during adolescence, although in recent years there has been an increase in new-onset EDs in adults [4, 5]. The lifetime prevalence of EDs in Western countries has been estimated to 1.89% [6]. Girls and women are more often affected than men. Previously, it has been estimated that about 90 percent of those affected are women, but new studies estimate that the proportion of men could be around 20 percent [7].

ED often require multi-disciplinary treatment [8]. Most patients are treated in outpatient care, but in more serious cases there may be both day care and inpatient medical or psychiatric care. There are also several inpatient units that specialise in treatments for patients with an ED [9].

The recommended psychological treatment for adult patients is cognitive behavioural therapy (CBT), which has is strongest empirical support for patients with bulimia nervosa (BN) and binge eating disorder (BED), but is also increasingly recommended for patients with anorexia nervosa (AN) [8–10]. Family-based treatment is the treatment method that is primarily recommended for adolescents. The method is mainly adapted for patients with AN or other restrictive conditions but is also considered to have a good effect for adolescents with BN [8, 9].

It is estimated that about half of all people with AN are fully recovered after treatment. At ten-year follow-up, about 73 percent are in remission. The short-term effect of treatment is slightly better for other types of EDs, but there is a significant risk of relapse. In ten years' time, there are marginally more people recovering from BN compared with AN [11].

Health care professionals often describe that patients with an ED are a challenging group of patients and that it can be difficult to establish a good treatment alliance [12]. Patients, on the other hand, often describe strong feelings of ambivalence and resistance, which of course complicates treatment, and leads to conflicts with family and friends [13, 14].

An improved common understanding of EDs from the perspective of those affected, their family members and caregivers can contribute to better care and treatment for those struggling with EDs and help reduce the strain on their relationships.

Against this background, the aim of the present study was to investigate experiences of living with an ED and factors that facilitate or hinder recovery from the perspectives of patients, their family members and health care professionals.

## Methods

The current meta-review is based on an assessment conducted at The Swedish Agency for Health Technology Assessment and Assessment of Social Services [15]. The literature overview was undertaken in accordance with the Enhancing Transparency in Reporting the Synthesis of Qualitative Research (ENTREQ) statement [16] following an a priori protocol that was registered locally at the agency.

### Search strategy

A systematic literature search covering literature published from January 1, 1990 to September 26, 2018, was conducted in the electronic databases PubMed (NLM), PsycInfo (EBSCO), Scopus (Elsevier), and CINAHL (EBSCO). A complementary, multi-database, search was also conducted. The databases Academic Search Elite, ERIC, Psychology and Behavioral Sciences Collection, and SocINDEX, were searched simultaneously through the EBSCO platform. The detailed search strategy is provided in Additional File 1.

### Eligibility criteria

Inclusion and exclusion criteria were specified in advance. We only included systematic reviews of qualitative research that were published in peer reviewed journals in English, Swedish, Norwegian, or Danish within the time period 1990 to 2018. To be included, a systematic review should cover experiences, perceptions, needs or desires related to EDs from at least one of the following perspectives: persons with eating disorders, family members or health care professionals. All types of EDs according to the DSM-5 classification were considered relevant except for pica, rumination disorder and avoidant/restrictive food intake disorder. There were no restrictions regarding the age of the informants. The targeted reviews were required to cover original studies of qualitative research or of mixed methodology. Systematic reviews using both broad and narrow search strategies were accepted. Grey literature, such as theses, book chapters, and conference abstracts, were excluded.

### Study selection

The titles and abstracts retrieved from the literature search were examined independently by two of the authors using the web-based screening tool Rayyan[17]. If at least one author found an abstract potentially relevant, the article was ordered in full text and assessed for eligibility by at least two authors independently. Systematic reviews that fulfilled the eligibility criteria were forwarded to quality assessment.

### Assessment of methodological quality, data extraction and analysis

Systematic reviews that fulfilled the eligibility criteria were assessed for quality by at least two authors independently, using a tool developed at the Swedish Agency for Health Technology Assessment and Assessment of Social Services (Additional File 2). The tool was developed specifically to assess methodological limitations of qualitative evidence synthesis and consists of 13 questions that were adapted from the ENTREQ recommendations [16]. The summarised risk of methodological limitations in the systematic reviews was judged as being of minor, moderate or high concern. Any disagreement between assessors was resolved by discussion. Systematic reviews with high concerns of methodological limitations were excluded from the subsequent process.

Relevant data were extracted from eligible systematic reviews with minor to moderate methodological concerns and summarised in tables.

The findings of selected systematic reviews (reviews with a narrow focus were not included in the synthesis) were analysed using the method of thematic analysis described by Braun and Clarke [18]. For each group of informants (patients, family members and health care professionals), findings were coded through an inductive analysis. Next, the coded findings were structured by subject within each group of informants and synthesised into themes. The themes were reviewed, similarities and disparities between the three groups of informants were analysed and the themes were assembled into main themes. Data extraction and synthesis was carried out by the first author (SAG) who has clinical experience treating EDs as well as expertise in qualitative research. Data extraction and synthesis of themes were carefully read and partly checked against the original data by three other authors who have experience of qualitative (AP) or quantitative research (KS, KWR) and expertise in conducting systematic reviews. The differing backgrounds of the authors presumably reduced the risk of introducing bias in the analysis and presentation of data. Throughout the synthesis, the authors discussed the findings with each other and reflected over how their background and position may have affected the analysis and whether there were other ways to interpret the results.

## Results

**Fig. 1:**
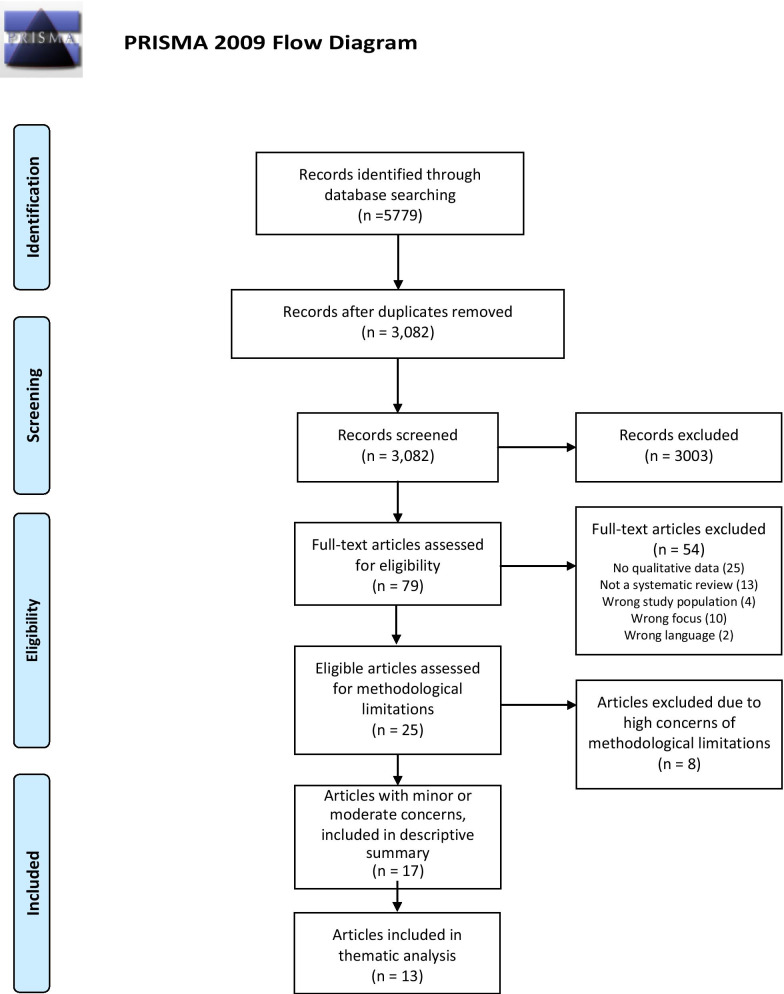
PRISMA flow chart

The literature search identified 3,082 citations, after removal of duplicates (Fig. 1). From the screening of title and abstracts, 79 reviews were retrieved and assessed for eligibility in full text, and 25 of these fulfilled our inclusion criteria. Eight reviews were considered to have high concerns of methodological limitations and were excluded from the subsequent process. The remaining 17 reviews were included and described (Table 1). Of these, four reviews had a scope that differed substantially from the other reviews (two reviews focused on pregnant women with AN [19, 20], one review focused on gender issues [21] and one focused on treatment seeking [22], therefore, they were only included in the descriptive summary but not in the thematic analysis. Thus, the thematic analysis included data from 13 systematic reviews.

**Table 1:** Included systematic reviews

**Author** **Year ** **Reference Country (first author)**	**Aim**	**Informants** **Perspective (patients, health care, relatives)**	**Included studies in total (number)** **Type of study **	**Method ** **Type of analysis **	**Author’s main conclusions**	**Concerns of methodological limitations**
Ali et al 2017 [22] Australia	**Aim** To systematically review the literature on perceived barriers and facilitators of help-seeking for eating disorders.	**Informants** ***Patient perspective*** Diverse groups of people with past or current ED or disordered eating. Most studies were based on community samples (mostly female) via advertisement.	**Included studies in total (number)** 13 studies **Type of study ** 3 quantitative 2 mixed 8 qualitative Studies published between 2001−2015.	**Method** Followed PRISMA **Type of analysis** Data analysis by Thematic Analysis (TA)	**Author’s main conclusions** Determining the factors that impede or facilitate help-seeking is critical in tackling ED. Reducing stigma and shame and educating people about ED, their impact and available resources is crucial.	**Concerns** Minor
Bezance et al 2013 [23] UK	**Aim** To review qualitative studies on the experience of treatment and recovery for adolescents with AN.	**Informants** ***Patient perspective*** Clinical samples (past or current) of adolescent and young adult patients with AN.	**Included studies in total (number)** 11 studies **Type of study** all qualitative or mixed method Studies published after 1950	**Method** No description of any method to ensure quality of included studies. **Type of analysis** Data analysis by Thematic analysis (TA).	Author’s main conclusions ** Patients described that access to specialist care was crucial although they reported both positive and negative aspects of specialist treatment, such as family therapy and inpatient treatment. The adolescents emphasised the need to address both psychological and physical aspects of the condition, to be fully recovered. Positive relationships with parents, siblings and friends had an important role in recovery.	**Concerns**Moderate Lack of information about if the researchers independently conducted the screening and appraisal with consensus
De Vos et al 2017 [34] The Netherlands	**Aim** To identify fundamental criteria for eating disorder recovery according to recovered individuals.	**Informants** ***Patient perspective*** Diagnostically diverse ED-samples (mainly female) who had recovered from an ED	**Included studies in total (number)** **Type of study** 18 studies all qualitative Studies published up to 4 February 2016.	**Method** Followed PRISMA Critical evaluation of studies according to CASP **Type of analysis** Data analysis according to Qualitative meta-analytic approach	**Author’s main conclusions **People who have recovered rate psychological well-being as a central criterion for ED recovery in addition to the remission of eating disorder symptoms. Supplementary criteria, besides symptom remission, are needed to measure recovery.	**Concerns** Moderate Not reported if two independent researchers conducted the appraisal with CASP
Duncan et al 2015 [25] Australia	**Aim** To enhance current understanding of recovery by synthesising the rich body of qualitative evidence examining the phenomenon from the perspective of those who have experienced it.	**Informants** ***Patient perspective*** Samples of recovered patients with AN (although some studies included even other diagnoses)	**Included studies in total (number)** **Type of study** 8 studies, all qualitative Studies published between 2003−2013.	**Method** Critical evaluation of studies according to CASP **Type of analysis** Data analysis by a Meta ethnographic approach.	**Author’s main conclusions **Recovery is described as a dynamic process involving a self-determined search for identity and truth and the repossession of personal control and power. The medicalisation of AN may downplay the wider human and social dimensions by the condition.	**Concerns** Moderate Lack of information about if the researchers independently conducted the screening and app raisal with consensus.
Eklund et al 2016 [24] Sweden	**Aim** To describe how eating disorders among adolescents affect family relationships and the family’s daily living conditions and to describe the family´s experienced need for professional support.	**Informants** ***Family member perspective*** Diagnostically diverse ED-sample and relatives (mainly parents) of adolescents suffering from an ED	**Included studies in total (number)** 15 studies **Type of study** 9 quantitative 6 qualitative Studies published between 2005−2015	**Method** **Type of analysis** Data analysis according to the Integrative method of Whittemore & Knafl [52]	**Author’s main conclusions** The group identity of the family is affected when an adolescent suffers from an ED, and emotional burdens on the family include isolation, and adapting to the situation. Input from health care professionals was crucial for the families. Parents’ experiences of the burden of care should take a central place when healthcare professionals discuss treatment options for the affected family.	**Concerns**Moderate Lack of information about if the researchers independently conducted the screening and appraisal with consensus
Espindola et al 2009a [27] Brazil	**Aim** To organize the body of information available in qualitative studies about the treatment of AN.	**Informants** ***Patient perspective*** Adolescent and adult patients (mainly female) in past or current treatment for AN (some studies had a mixed sample) according to DSM-IV criteria. Two studies also included some participants with no treatment.	**Included studies in total (number)** **Type of study** 15 studies, all qualitative Studies published between 1990−2005.	**Method** Study quality assessed according to CASP **Type of analysis** Data analysed by a Meta ethnographic approach	**Author’s main conclusions** Recovery from AN, as a very complex process, goes well beyond conventional treatment. Self-acceptance, determination, and spirituality are equally important elements.	**Concerns** Moderate Lack of information about if the researchers independently conducted the screening.
Espindola et al 2009b [28] Brazil	**Aim** To develop a hypothesis about the nature of AN and how it relates to more effective therapeutic interventions.	**Informants** ***Patient perspective*** Adolescent and adult participants (mainly female) with past or current AN (some studies had a mixed sample) according to DSM-IV criteria	**Included studies in total (number)** **Type of study** 24 studies, all qualitative Studies published between 1990−2005	**Method** Study quality assessed according to CASP **Type of analysis** Data analysed by a Meta ethnographic approach	**Author’s main conclusions** Knowledge of patients ‘efforts to interpret the illness as a part of their own identity and sense of control have a key role in in physician understanding of the disorder by allowing physicians to bring structure to the patients’ lives generally and to their help-seeking behaviour specifically.	**Concerns** Moderate Lack of information about if the researchers independently conducted the screening and appraisal with consensus.
Espindola et al 2009c [26] Brazil	**Aim** To carry out a systematic review on how family members perceive AN and bulimia nervosa patients.	**Informants** ***Family member perspective*** Family members of a mixed ED sample (mainly adolescent or young adult women with AN). Some studies also included patients, but only data from the relatives were included in the analyses.	**Included studies in total (number)** **Type of study** 9 studies (from a total of 7 study populations), all qualitative Studies published between 1990−2006.	**Method** Study quality assessed according to CASP **Type of analysis** Data analysed by a meta-ethnographic approach.	**Author’s main conclusions** Care provided to patients should include the opportunity of examining and consulting family members, give clarification and information about patient care, and situations involving pathological functioning of patients and their family. Support networks and self-help networks such as meeting with families experiencing similar situations should be considered.	**Concerns** Minor
Fogarty et al 2018 [19] Australia	**Aim** To examine the experience of women with an eating disorder in the perinatal period: that is during pregnancy and two years following birth.	**Informants** ***Patient perspective*** Mixed ED sample of women that were pregnant or in the perinatal period.	**Included studies in total (number)** **Type of study** 12 studies, all qualitative Studies published later than 1980 was considered.	**Method** Study quality assessed according to CASP **Type of analysis** Data analyzed by a Meta ethnographic approach.	**Author’s main conclusions** Following a tumultuous pregnancy experience, many described returning to their pre-pregnancy eating behavior and thoughts, which highlights the emotional difficulty of having an ED whilst pregnant, but also points to opportunities for intervention and a continued acceptance of body image changes.	**Concerns** Minor
Fox et al 2017 [29] UK	**Aim** To synthesize qualitative studies relating to the caring experience and its impact, thereby gaining an understanding from the perspective of the individuals themselves.	**Informants** ***Family member perspective*** Most participants were parents, but some studies also included partners and siblings. Most participants had a family member diagnosed with AN, but some studies also included relatives of patients with BN.	**Included studies in total (number)** 20 studies **Type of study** 1 mixed 19 qualitative Studies published after 1970	**Method** Study quality assessed according to CASP **Type of analysis** Data analysis based on metasynthesis according to the principles of Noblit & Hare [47]	**Author’s main conclusions** The ED was found to have a pervasive impact upon family members, mediated by a number of factors. Cognitive appraisals affected the caregiving experience and responses to the individual. The experience of caregiving was continually reappraised leading to a process of adaptation. Most of studies identified unmet career needs.	**Concerns** Moderate Lack of information about if the researchers independently conducted the screening and appraisal with consensus.
Medway et al 2016 [30] Australia	**Aim** To describe patient´s experiences of family interventions for AN.	**Informants** ***Patient perspective*** The sample included patients (mainly adolescents) with a current or former diagnosis of AN who had underwent a therapist delivered family intervention. Some studies had a mixed ED-sample	**Included studies in total (number)** **Type of study** 15 studies, all qualitative Studies published up to November 2015.	**Method** Critical evaluation of studies according to COREQ **Type of analysis** Data analysis based on metasynthesis using thematic synthesis according to Thomas & Harden, 2008.[48]	**Author’s main conclusions** Strength of family-based approaches included support of family understanding and use of the family as a resource for recovery. Addressing a variety of underlying family and individual issues was implicated as an area for improvement.	**Concerns** Minor
Salzmann-Eriksson et al 2017 [35] Sweden	**Aim** To identify and describe factors that promote and impede the relationships between nurses and children, adolescents and young adults who are diagnosed with AN and also to explore and describe how those relationships benefit the patients’ process toward increased health and well-being.	**Informants** ***Health care perspective*** A sample of adolescent and adult patients with AN and nurses (both in general and specialised care) in mainly inpatient treatment for AN.	**Included studies in total (number)** **Type of study** 14 studies, all qualitative Studies published between 2004– 2014.	**Method** Quality of included studies was assessed based on a review template published by Forsberg & Wengström, 2013 [49] and Willman et al., 2006 [50 ] **Type of analysis** Data was synthesised through the process outlined by Evans, 2002 [53 ].	**Author’s main conclusions** Nurses need to be person-centred in their relationships with patients and to have attitudes characterised by presence, genuine commitment and motivation. Nurses are more likely to convey a sense of trust and safety when they communicate with openness and honesty.	**Concerns** Moderate Not reported if two independent researchers conducted the appraisal with CASP.
Sibeoni et al 2017 [32] France	**Aim** To perform a systematic review of qualitative studies to synthesize the views of adolescents with AN, their parents, and their healthcare providers about its treatment.	**Informants** ***Patient perspective, family member perspective and health care perspective*** Participants could be patients (younger than 18 years during their disease, AN), their families, or the healthcare professionals caring for them.	**Included studies in total (number)** **Type of study** 32 studies, all qualitative Studies published between 1990– 2014.	**Method** The study complies with the ENTREQ guidelines, which includes critical evaluation of study quality according to CASP **Type of analysis** Data analysed by a Meta ethnographic approach	**Author’s main conclusions** The results underline the difficulty in establishing a therapeutic alliance, the barriers to it, especially the risk that professionals, adolescents, and parents will not converse about treatment; although such a dialogue appears to be an essential component in the construction of a therapeutic alliance.	**Concerns** Minor
Sibeoni et al 2017 [31] France	**Aim** To explore how AN is experienced by adolescents, their families and the health care professionals who provide care for them and to compare their perspectives.	**Informants** ***Patient perspective, family member perspective and*** ***health care perspective*** Study samples included people who had experienced having AN during adolescence (younger than 18 years during their disease), parents of adolescents with AN, and health care professionals with experience in the field of AN.	**Included studies in total (number)** **Type of study** 30 studies, all qualitative Studies published between 1990–2015	**Method** The study complies with the ENTREQ guidelines, which includes critical evaluation of study quality according to CASP **Type of analysis** Data analysed by a Meta ethnographic approach and follows the procedure of thematic synthesis by Thomas & Harden, 2008 [48]	**Author’s main conclusions** There were important disparities between three different stakeholders. The adolescents underlined the psychological and emotional aspects of their experience, while the visible state of these patients’ bodies impeded the work of the professionals. Treatment of AN in adolescence must integrate both psychological and physical components.	**Concerns** Minor
Stockford et al 2018 [33] UK	**Aim** To systematically review qualitative studies which have investigated female service users’ experiences of recovering from AN.	**Informants** ***Patient perspective*** Study samples of adolescent or adult individuals who had fulfilled DSM-IV or DSM-5 criteria of AN. The majority of informants were recovered or in various stages of recovery during the data collection.	**Included studies in total (number)** **Type of study** 14 studies, all qualitative Studies published between 2002–2017	**Method** Study quality assessed according to CASP **Type of analysis** Data analysed by a Meta ethnographic approach.	**Author’s main conclusions** Recovery from AN is experienced as a complex psychological process with many contributing factors. Findings highlight the need to reconsider clinical practice and treatment provision to incorporate the psychological components of self-identity into recovery programs.	**Concerns** Moderate Lack of information about if the researchers independently conducted the screening and appraisal with consensus
Thapliyal et al 2018 [21] Australia	**Aim** To more richly understand issues related to gender in EDs and their treatment across relevant qualitative research studies.	**Informants** ***Patient perspective*** Study samples included participants of all ages and genders that had an ED according to current diagnostic schemes. One study also included representatives of organizations and health care practitioners.	**Included studies in total (number)** **Type of study** 9 studies, all qualitative Studies published between 1980–2017.	**Method** Study quality assessed according to CASP and RATS **Type of analysis** Data analysis according to principles of Shaw, 2012.	**Author’s main conclusions** Gender issues impact upon the ED experience and require broader consideration in the development and evaluation of ED treatment interventions, including the further development of gender-informed interventions.	**Concerns** Minor
Tierney et al 2013 [20] UK	**Aim** To synthesise qualitative studies that focused on the perspective of women with an ED in relation to being pregnant	**Informants** ***Patient perspective*** Women with self-reported or diagnosed ED that were pregnant or had given birth at the time of data collection	**Included studies in total (number)** **Type of study** 7 studies, all qualitative Studies published from 1980 and onwards.	**Method** Study quality assessed according to CASP **Type of analysis** Data analysis by framework analysis (Ritchie et al., 2003) [51]	**Author’s main conclusions** Participants reported vacillating between wanting to do the best for their child, being motivated by social pressures and feeling the need to control their body for self-preservation purposes. This created the inner turmoil they experienced while pregnant.	**Concerns** Minor

### Descriptive summary of the systematic reviews

The 17 systematic reviews with minor or moderate concerns of methodological limitations were published between 2009 and 2018 and were based on a total of 255 unique qualitative primary studies. An assessment of study overlap revealed that few of the primary studies were included in more than one review (see Additional File 3). The majority of the included reviews were based on studies using qualitative methods only, but three reviews also included studies that used mixed methods [22–24]. Most of the original studies had used interviews as the primary source of data, but some studies were based on focus group discussions, survey responses, or observations of behaviour.

Most reviews carried out synthesis using meta-ethnography [19, 25–33]. Other synthesis methods were thematic analysis [22, 23], qualitative meta-analysis [34], and various forms of integrative synthesis methods [20, 21, 24, 35]. Few of the included reviews stated that they had followed the Preferred Reporting Items for Systematic Reviews and Meta-Analysis (PRISMA) statement[22, 34] or the Enhancing Transparency in Reporting the synthesis of Qualitative research (ENTREQ) system[31, 32]. In most reviews, however, the Critical Appraisal Skills Programme (CASP) had been used to assess the quality of the primary studies [19–21, 25–29, 31–34].

A total of 13 systematic reviews described the patients’ perspectives [19–23, 25, 27, 28, 30–34], five concerned the family members’ perspectives [24, 26, 29, 31, 32], and three focused on the health care professionals’ perspectives [31, 32, 35]. Most reviews included both men and women with EDs, and only three reviews focused exclusively on women [19, 20, 33]. Most reviews did not specify age under the inclusion criteria, but no review included studies on young children. Five reviews focused on adolescents with EDs but they also included young adults [23, 24, 30–32]. Nine reviews focused exclusively on AN [23, 25, 27, 28, 30–33, 35], while the remaining reviews included all EDs, or did not specify diagnosis in the inclusion criteria. One of the reviews that covered health care professionals’ perspectives included interviews exclusively with nurses [35] whereas the other two comprised nurses, therapists, and treatments teams [31, 32]. The informants in the reviews that included family members were predominantly parents, but siblings and partners were also included in some of the reviews [24, 26, 29, 31, 32].

### Thematic analysis of the systematic reviews

In each of the three perspectives we identified three themes that described experiences of the disease, the care provided and the recovery process. When the three perspectives were analysed together, we identified three overarching themes that were shared among all three perspectives (see Table 2). The themes are described in the table below and organized by perspective. Illustrative quotes for each theme are provided in Table 3.

**Table 2:** Overview of subthemes and overarching themes from the 13 systematic reviews that were included in the thematic analysis

Patients with ED	Health care staff	Family members	Overarching theme
A lonely struggle for control	A tug of war over control	The balancing act between control and trust	Being in control, or being controlled
A wish to be seen as a whole person	The necessity of physical recovery	A call for a more holistic approach to treatment	Balancing physical recovery and psychological needs
Finding the keys to recovery	Being let in to someone’s world	A wish for a working alliance with the whole family	Trusting relationships

**Table 3:** Themes and quotations from the systematic reviews

Overarching theme	Subtheme	Illustrative quotation
**Being in control, or being controlled** Illustrative quotation for overarching theme: "Most adolescents with anorexia nervosa placed themselves in a dialectic of both controlling and being controlled. […]They reported a positive feeling of self-control […] but also in their relationships, in particular with healthcare professionals […]. Nonetheless, they also described the distressing feeling of being controlled or trapped by the disease and of losing control […] For parents, anorexia nervosa was a disease that has taken control of their child and modified his or her behaviour […]. It also affected family relationships, well-being, and daily life […] and it created a feeling of insecurity within the family and mistrust in intrafamily relationships and communication […] Healthcare providers perceived that the adolescent's search for control was at the heart of anorexia nervosa […] specifically the need to control others, especially the family". [31]	*Patients with ED* A lonely struggle for control	“In many accounts anorexia nervosa is described as something that provides safety and protection on several levels. ‘Anorexia nervosa, my friend… You’re my source of safety, my guardian…’ […] Loss of weight is seen as a remarkable conquest and as a sign of extraordinary personal discipline, whereas weight gain is considered an unacceptable failure of self-control. Not eating gives patients a sensation of control over their own lives. They feel stronger when they do not eat and totally in control of the situation. “you can have control on all your body, you can do things that other people say you can’t.’" [27] [p.75] "Engagement in anorexic behaviours was consistently reported as a way for the individual to gain control in their lives. Ironically, as the condition worsened, the self-imposed stringent rules […] resulted in the participants feeling even less control in their lives” [25] [p.182] “Thus, anorexia nervosa passes from an effort to attain control to an entity controlling their lives. ‘It’s like a monster… something that holds you with its claws." [27] [p.75]
		"Patients struggled with allowing others to take control with respect to their eating-related behaviours. At the same time, however, they often appreciated that this was necessary to recovery." [30] [p.197]
	*Health care staff* A tug of war over control	“Lack of knowledge could result in a perception among nurses that the patients themselves were responsible for the illness and hence should be able to ‘fix themselves’[…] Such attitudes toward eating disorders entailed that the nurses performed routine behaviour and control work. […] As a consequence of nurses’ lack of knowledge about anorexia, the acute divest of patients’ control of meals resulted in power structures that extended into other areas of the patients’ lives.” [35] [p.9} "For most professionals, the therapeutic relationship […] included an aspect of control. They considered it necessary to assume control of the adolescents’ actions to enable normalization and the disappearance of symptoms. They believed that they must decide in the patients’ place […] and maintain a framework, structured by the department’s rules and protocols […]. Some professionals tried to balance their controlling approach with kindness […] but, most of the time, this takeover induced a power struggle." [32]* [p.12]*
	*Family members* The balancing act between control and trust	“during this disharmonic state, the roles, rules and relationships within the family change and control the family’s everyday life.” […] Parents describe that the illness controls and takes over the discordant family, which creates an unpleasant climate”. [24] [p.220] “constructing the ED as a separate entity […] included actions whereby carers were ‘tough’ on the ED, but ‘kind’ to the individual.[…] Difficult behaviours and negative emotions were attributed to the ED, enabling carers to remain empathic yet resist the wishes of the individual to promote recovery. […] Once you separate you can fight it. While you’re seeing it as being one you can’t fight yourself, it made things here a lot easier because once I could differentiate between the two of them; and then you would say, is this you talking or her?” [29] [p.115]
**Balancing physical recovery and psychological needs** Illustrative quotation for overarching theme: “the healthcare professionals […] relied on a biomedical discourse to define the target symptoms and their normalization. […] From the point of view of the professionals, treating AN was equivalent to normalizing the patient’s weight, body, and behavior. […] Retrospectively, some adolescents recognized the importance of regaining weight and changing their behavior […] but most of them criticized the method used and its effects. They denounced the use of the criterion of weight alone to judge health status and the course of care […]. They also considered that the treatment focused too much on somatic aspects, while ignoring their psychological distress […]. Parents shared this opinion and regretted that care focused too much on their child’s physical health. [32]"	*Patients with ED* A wish to be seen as a whole person	“ distinction between the physical symptoms of anorexia and the psychological aspects of the condition in their treatment and recovery. […] adolescents lost their sense of identity as staff conveyed assumptions about how ‘an anorexic’ thinks and behaves. This non individual approach was also mirrored in the use of standardised treatment programmes and a focus on physical recovery over psychological recovery where the treatment goal was ‘to fatten them up’." [23] [p.356] “An eating disorder does not disappear just because you start eating right.[…] the treatment of anorexia nervosa is not exclusively a question of weight and eating habits. Nutritional treatment designed to increase weight, which may at times involve the imposition of certain rules, is understood to be unsatisfactory, in that no consideration is given to the psychological aspects of anorexia nervosa, nor does it provide emotional support to the patient." [27] [p.44] “Patients found that family therapy neglected some important issues […] patients believed that the causes of AN were neglected in treatment, and would have liked attention to this. […] Some participants receiving FBT expressed that they would have appreciated issues other than AN being addressed in therapy. […] an important perceived shortcoming of ‘family counselling’ was that ‘personal problems/feelings’ were neglected in favour of focusing on eating behaviours." [30] [p.200]
	*Health care staff* The necessity of physical recovery	“This theme […] was predominant in the healthcare professionals' representation of treatment, for they relied on a biomedical discourse to define the target symptoms and their normalization. Professionals considered AN, which they viewed as a disease or disorder to be corrected, as the object of treatment. […] From the point of view of the professionals, treating AN was equivalent to normalizing the patient's weight, body, and behaviour.” [32] [p.11] “Several studies stressed the nurses’ inability to pay attention to patients’ needs for psychological support to the same extent as they did to the physical issues. Such a single minded focus on weight reinforced the feelings among patients of being their diagnosis. […] The unbalanced focus hampered the relationship as patients perceived that the nurses did not want to be supportive of all their needs but, rather, saw only the goal of the patients reaching a certain weight […]). The feeling of loss of control due to weight gain and lack of psychological support strengthened the anorexic behaviours, thereby working against the patient’s health process” [35] [p.8]
	*Family members* A call for a more holistic approach to treatment	"Parents considered this biomedical theory of anorexia nervosa and the treatment framework it implied to be too rigid and to prevent professionals from caring for their child´s global distress." [31] [p.30] “…above all, care should focus on the adolescents as individuals and complete people […]. This holistic approach distinguished three aspects: the teen´s involvement in their own care, consideration of their social world, and consideration of their families." [32] [p.11] “In all studies participants reflected on meaningful relationships with others as being an integral component of reclaiming a sense of self in their journey to recovery. Relationships, whether with partners, family, friends, others with an ED or therapists, enabled women to learn to accept themselves through the experience of acceptance by others.” [33] [p.20]
**Trusting relationships** Illustrative quotation for overarching theme: “ all considered the therapeutic relationship as the core concept for ensuring the effectiveness of treatment. Indeed, they had the same vision of the benefits of a good therapeutic relationship and about the conditions for constructing a therapeutic relationship. […] they experienced the same barriers to establishing a good therapeutic relationship: mutual distrust and lack of communication.” [p 13] For health-care providers, establishing a therapeutic relationship […] was the major challenge […]. The professionals considered relationships with the parents important as well […] Parents reported that three actions appear necessary to guarantee a trusting relationship between the professionals and themselves: the professionals must support them […], involve them […], and inform them. [32]	*Patients with ED* Finding the keys to recovery	“reducing disconnection from others, particularly family, was a key aspect of addressing the difficulties underlying AN, and therefor in promoting recovery." [30] [p.198] “Staff who were sensitive to individuals and their needs, for example, empathetic, clear, consistent and who demonstrated availability and willingness to listen, were felt to be crucial in treatment and recovery.” [23] [p.356]
		“Where staff were empathetic and non-critical, psychotherapy imparted hope and facilitated self-determination whilst allowing participants to feel safe, supported, and validated.” [25] [p.184]
	*Health care staff* Being let in to someone’s world	“…actively allowing the patients to be more involved to create an alliance. […] lack of involvement could lead to resistance and hamper the recovery process and the relationship and could even worsen the illness. […] The nurse’s ability to establish a personal connection in the relationship was described as important in the recovery process and essential for the establishment of a trusting relationship. [35] [p.7] "The aspects of openness, integrity and honesty were identified as vital in establishing a relationship […] the feeling that they were not just ‘doing a job’, contributed to a sense of safety among the patients. […] it was only when the nurses really showed a genuine commitment that patients felt meaning in care. Such commitment made it possible for the patient to see beyond the role of the nurse only as a professional, which promoted an individual and unique relationship. […] On the contrary, in situations when nurses failed to demonstrate such commitment and genuineness, it resulted in a sense of ‘us versus them’” [35] [p.7]
	*Family members* A wish for a working alliance with the whole family	“Several studies highlight the importance of involving the family as a whole in the treatment and use of psycho-education in order to increase the family’s knowledge about the illness. […] In order to establish structures that the family can rely upon, it is necessary that both parents and children work together against the illness..” [24] [p.223] “family members of patients revealed unaddressed needs such as information and practical guidance on how to manage the patient for they felt impotent and needed to share this experience with other people. […] “We need guidance on how to manage daily situations, this is my main problem now…..if she has an eating binge, what should I do? Should I try and stop her? Should I try to talk to her? Or should I distract her…” [26] [p.4] “…the majority of carers described feeling excluded or ´shut out´ of treatment. Often this exclusion was attributed to confidentiality, legislation, which created a perceived ´wall of silence´, leaving carers feeling uninformed regarding the individual´s treatment and prognosis." [29] [p.119]

#### The perspective of individuals with ED

Nine systematic reviews describing the patients’ perspectives were included in the thematic analysis [23, 25, 27, 28, 30–34]. This perspective comprised three themes; a lonely struggle for control (covered by three studies [28, 31, 33]), a wish to be seen as a whole person (covered by four studies [23, 27, 30, 32]), and finding the keys to recovery (covered by five studies [23, 25, 27, 33, 34]).

### A lonely struggle for control

Life with an ED was described as a lonely and isolated existence, with health problems and difficulties in relationships [28, 31, 33]. Low self-esteem, a negative body image and perfectionist demands on themselves were seen as underlying factors that led to a difficult adolescence, and uncertainty about who they were.

For those with AN, the disorder was seen as an integral part of their personality and the person they were, which also made them afraid to get well since they feared that it could mean losing their identity [28, 31, 33].

Living with AN was described as a struggle to be in control while simultaneously feeling controlled by the disease. The positive experience of control contributed to feeling special and having power (for example over their treatment) and the ED was described as a "coping strategy" that helped them deal with difficult emotions and events. For the majority of patients, the other side of the coin was a difficult experience of losing, or giving up control, for example when entering treatment, or feeling trapped in their illness and symptoms. The subjects described how their whole life revolved around a compulsive focus on calorie counting and compensatory behaviours and how this resulted in a lonely and isolated existence [28, 31, 33].

### A wish to be seen as a whole person

When seeking treatment, patients had often felt ill-treated and misunderstood, especially in general care [23, 27, 30, 32], and therefore, they stressed the necessity of access to specialised ED care. The patients often felt that the health care focused too much on physical recovery and on normalization of eating and weight. This was perceived as unempathetic and gave patients the impression that the therapists did not understand the patient's real problems. Although patients could see that normalization of weight and eating was an important and necessary part of treatment, they felt that focusing too heavily on physical recovery led to feel that they were being reduced to their disease [23, 27, 30, 32].

Instead, they emphasised that there must be room for conversation about thoughts and feelings and that the care they received should take their wider life situation into account. It was also felt necessary that the therapistwas able to adapt and change his/her approach during the course of the treatment. Initially, the patients might need a therapist that was proactive and took control. At a later stage when the patient was able to take responsibility, treatment should empower and encourage the patient to take control of his/her own life.

Family-based treatment was common for young patients. These patients often felt considerable guilt towards parents and siblings, and they described that a positive aspect of family treatment was that it could help the whole family to feel better, bring them together, and improve their communication. However, patients also described feeling unable to talk about everything that was important to them in family treatment. This risked the treatment becoming superficial and focused on concrete behavioural changes instead of dealing with the underlying causes of the condition. The young patients therefore felt that it was important that family treatment was combined with individual therapy. Individual therapy was seen as an important forum for motivating, engaging and giving patients hope. Patients perceived that it was important to address issues such as relationships both within and outside the family, and to be seen as a unique individual, rather than simply as a person with AN [23, 27, 30, 32].

### Finding the keys to recovery

In the studies that focused on AN, patients consistently described recovery as something “greater” than the mere absence of an ED diagnosis. An experience of being healthy did not arise automatically once weight and eating were normalized. Patients described recovery as a process of getting to know themselves and daring to admit that the false sense of control that the ED had given them had actually come to control them. Recovery meant being able to stick to healthy behaviors even when it felt difficult [23, 25, 27, 33].

Four factors were described as central to recovery; to regain control and power over one's own life, changing the anorexic identity and finding and accepting oneself behind the disease; getting in touch with one's true feelings and acknowledging the consequences of the disease for oneself, thereby challenging the anorexic thoughts.

In the systematic review that described recovery more generally for people with an ED, it was found that patients perceived the term "healthy" as including feeling well emotionally, socially and psychologically. It included having strategies for dealing with difficulties that arise in life and feeling a sense of belonging or feeling that life is meaningful.

Recovery was described as a process that took place in stages and sometimes with setbacks. Recovery was facilitated by supportive relationships, such as with family and friends. Trusting relationships with family and friends could have a double impact, both by motivating the ill person to seek treatment [25] and by providing support during the recovery process. Trusting relationships with health care professionals were also considered important both for the motivations to seek and stay in treatment [25] and for the recovery process itself [34].

### The health care perspective

Three systematic reviews included the experiences of health care staff [31, 32, 35], and all three focused mainly on AN. Two of them [31, 32] examined similarities and differences in perceptions of AN and its treatment among staff, patients and relatives. The third overview [35] explored the knowledge, attitudes and perceived challenges of health care professionals.

The health care perspective also revealed three themes: a tug of war over control, the necessity of physical recovery, and being let into someone’s world.

### A tug of war over control

The health care staff saw control as a central aspect of AN and they felt that besides the need to control their own body, patients also felt a need to control their family through the ED [32]. The staff perceived that the need for control became a force outside the patient's active choice and that the ED ended up controlling the patient instead. The staff therefore felt that they had to "take over" control from the young person through clear structure and rules regarding treatment [32, 35]. This was considered to create security for the young person, and to give them the opportunity to allow themselves to let go of control. However, in one study, nurses also stressed the importance of knowledge and understanding of the disease, and described how a lack of knowledge could lead to staff using control strategies in a repressive and punishing way that could create resentment [35].

### The necessity of physical recovery

The health care staff used a biomedical model to understand AN [31, 32]. AN was seen as a disease to be treated. This meant that staff emphasised weight rehabilitation and changes in other observable ED symptoms as important parts of treatment. The staff expressed that they were lacking knowledge about ED symptoms and diagnosis, and that they had insufficient skills for dealing with patients' problems [35]. This led them to feel frustrated and insecure in meeting the patients. Increased knowledge was seen as essentialfor improving staff attitudes towards people with EDs.

The medical view of the ED was perceived as helpful by staff because it was considered to reduce the patient's and their relatives' feelings of guilt. Health care professionals found it helpful to see the disease as a phenomenon separate from the individual. The staff used this "externalisation" to distinguish between disease and patient as a treatment strategy [31, 32]. It was considered to reduce the patient's feelings of guilt and increase the patient's motivation.

Even under this theme, a review by Salzman et al. [35] also emphasised the other side of the coin, meaning.e., that although weight rehabilitation was important, a single-minded focus on physical issues could hamper the relationship with the patient.

### Being let in to someone’s world

A good alliance between patient and therapist was considered essential [31, 32, 35]. Honesty, understanding, respect and a non-judgmental and empathetic attitude were important for building an alliance. The staff expressed that patients with EDs were a difficult and demanding patient group with whom it was challenging to form an alliance and who often expressed suspicion and distrust of their caregivers. Staff became frustrated with patients' ambivalence or reluctance to engage in treatment and sometimes perceived patients as manipulative.

One of the systematic reviews examined the health care professionals' experiences of meeting relatives, in this case parents of people with an ED [32]. The staff emphasised the importance of building a positive alliance with the parents and engaging them in the treatment. This was considered a necessary condition for effective treatment of young patients with AN.

### The perspective of family members

Five systematic reviews covered the perspective of family members [24, 26, 29, 31, 32]. All of them focused mainly or exclusively on AN. Like the other two perspectives, the perspective of family members also revealed three themes; the balancing act between control and trust, a call for a more holistic approach to treatment, and a wish for a working alliance with the whole family.

### The balancing act between control and trust

The family members felt that the whole family was negatively affected by the afflicted person’s illness [24, 26, 29, 31, 32]. The family members described the ED as an active choice which the sufferer, at least at some point during the course of the disease, could have refrained from [29]. The family members felt that controlling eating and weight had, for the ill person, become a way of coping in a life where other things felt uncontrollable, but that the ED had instead taken control of their loved one and changed her personality and behaviour [26, 29, 31]. Family patterns and old roles changed [24, 26, 29, 31, 32] and the family members described communication as characterised by conflict, mistrust and uncertainty. It could be perceived that the person with the ED had regressed, which led parents to become more controlling. The opposite sometimes happened with siblings, who would take on a more mature role, becoming a "mediator" in the family and taking greater responsibility.

Family members described a difficult balancing act between adapting to the ill person by, for example changing the family's eating habits and activities, andbeing more demanding. The family members tried to find a balance between controlling and making demands on the ill person, and at the same time reinforcing and encouraging positive steps and showing trust in her/him. To some extent, they felt that it was important to adapt the family's social activities and meals by, for instance, not having certain foods in the house. However, this sometimes resulted in them "walking on eggshells" and accepting behaviours that were counterproductive in the long term. Siblings were often critical of the parents' strategies and thought that they adapted too much.

A common strategy to cope with this balancing act was to distinguish the disease from the individual and to see certain behaviours as “the disease that speaking”. This helped the family members to maintain a supportive attitude, even when they felt that the person with the ED was misbehaving [24, 26, 29, 31, 32].

### A call for a more holistic approach to treatment

It was stressful to see the person with an ED suffering, and the family members felt anxiety, frustration and guilt. Their everyday lives were affected, both socially and professionally. Many informants reported that the family became more isolated and that they stopped associating with others. Several of the systematic reviews reported that family membersno longer had time for hobbies and that working life was affected [24, 29]. Against this background, family members stressed the importance of easier and faster access to specialised care with experienced and committed staff who could give the whole family including siblings information and support, and put them in touch with support networks outside the family to connect with others who were in the same situation. [24, 26, 29, 31, 32].

Parents often felt that the health care model was too biomedical and focused too much on physical symptoms such as starvation. They perceived that the unique person behind each patient was not seen [24, 29, 32]. Although the biomedical explanatory model could help to relieve parents' feelings of guilt, it also conveyed a negative image of the patient's chances of recovery [29]. The family members emphasised that it was important that the therapist saw the patient as an individual and that the therapy did not focus too narrowly on correcting the ED symptoms, but also incorporated other things that were important to the patient [24, 29, 32].

### A wish for a working alliance with the whole family

The parents often blamed themselves for their child’s ED [24, 26, 29, 31, 32] and they thought a lot about it’s possible origins in the family and the child’s upbringing. The siblings felt severely affected by the situation, something that was also described by their parents [24, 26, 29, 32]. Siblings became anxious and often took great responsibility for both the afflicted sibling and their parents. At the same time, they often felt angry with their unwell sibling, and sometimes jealous that they were receiving more time and attention from their parents. The healthy siblings sometimes felt a conflict of loyalty and also were compelled to mediate between the afflicted sibling and the parents [29].

Family members often experienced a lack of support from the health service, especially at the beginning of the illness [24, 26, 29, 31, 32]. It was difficult to get a correct diagnosis and adequate help, and family members had to fight to get the right care for the affected person. Family members often felt excluded from care and experienced that health care staff did not support them or listen to them. This exclusion was often attributed to rules or principles that had to do with confidentiality or legislation. Family members also felt that they received conflicting advice and suggestions from the health service or that they were not taken seriously [29].

### The three overarching themes

Our synthesis identified three themes in common among the views of patients, family members and health care professionals (Table 2). The first theme pertained to the patients’ need for control, which was seen by the family members and the health care professionals as a false control, where the affected person was in fact controlled and limited by the ED. The second theme was the balancing of physical recovery and psychological needs, where the biomedical model was viewed differently from each of the three perspectives. Health care professionals felt that, if used with the right knowledge and competence, the model gave them the support they needed to define target symptoms and goals for recovery, while patients and family members felt that the model placed too much focus on the somatic aspects of the disorder and failed to address psychological distress. The third theme was the importance of forming trusting relationships for accomplishing a well-functioning therapeutic alliance that recognises the whole individual and not just the disease, and that also involves family members.

## Discussion

This meta-review brings together a substantial amount of qualitative research, including data from 255 unique studies, on the experiences of EDs from the perspectives of patients, family members and health care professionals. Three themes emerged from the synthesis; the patients’ need for control, balancing physical recovery and psychological needs, and the importance of trusting relationships in the treatment of the disorders. Although all three main themes were identified in the views of all three groups of informants, there were some differences in their expression that may be important to acknowledge.

### Implications for health care systems

The ED causes a great deal of suffering for both the affected person and the family members, and both parties emphasise the importance of getting the right treatment. From our synthesis, however, there appears to be a divergence between ED patients and their family members on the one hand, and the health care staff on the other, regarding how the ED should be understood and treated. Health care professionals often represent a biomedical explanatory model, while ED patients and their family members feel that this model is not sufficient. These different approaches are not necessarily conflicting, but can potentially complicate the alliance building and pull the treatment in different directions, where the professionals place more emphasis on symptom reduction and weight rehabilitation, while the patients and their family members want a more holistic approach to treatment and recovery. This conflict, and suggestions for how to avoid it, was also emphasised in one of the studies involving health care staff [35]. The main suggestion from patients, family members and health care staff on how to achieve this holistic approach, while still attending to the physical needs of the patient, was to increase the knowledge. The importance of having access to staff who are knowledgeable in terms of both understanding the disease and attending to the patient’s physical needs, and understanding their psychological struggles, and are able to meet the patient in a respectful way, cannot be overemphasised.

In today's health care, and among policy makers, there is an increasing focus on using manual-based treatments and on measuring the outcomes of treatment. Great emphasis is placed on questions about which treatment method has the best scientific support, and how to make sure that therapists actually deliver the method according to the manual [36]. These are of course important questions that need to be addressed. However, it is important to acknowledge that these aspects seem to be entirely absent from patients’ and families’ descriptions of what is lacking or what is important in treatment. On the contrary, persons with an ED's desire treatment that is more flexible and individualised, with greater focus on their unique, individual situation. None of the systematic reviews in this study mentioned that patients or family had called for any specific method of treatment, instead they called for a more holistic and individually-adapted care. Since a significant proportion of ED patients discontinue treatment prematurely [37], and a common reason for this is lack of motivation [38], it is important that health care providers increase their knowledgeabout how patients and family members perceive the care provided, and what would motivate patients to stay in treatment.

Treatment manuals are a set of principles designed to be applicable to each individual patient. When delivered flexibly and skilfully there is no reason why individualised care should be in conflict with the use of treatment manuals [39]. However, many clinicians regard treatment manuals as constraining their practice and limiting the individualisation of interventions [39]. Against this background, and the findings of this study in terms of patients and relatives calling for a more holistic and individualised treatment, it seems that ED treatment faces a great challenge in integrating theory, research, clinical knowledge and the important perspectives of patients and their families in order to improve and adapt ED treatment. For this to be successful, it has been suggested that we need to expand the scope of treatment research and stimulate diversity within ED treatment and research [40].

### Limitations and strengths

One limitation of this meta-review, which is a common problem in qualitative research syntheses, is the considerable variability in research aims, data collection approaches and methods of synthesis that were present in reviews as well as in the primary studies. Another problem that is difficult to avoid in qualitative syntheses is the possibility that the authors’ underlying assumptions may have introduced bias through selection of the experiences and views that are presented in the studies. The risk of overestimating the findings through data redundancy should also be considered, but is probably not a major problem in this meta-review since most of the included reviews had a unique focus and the study overlap was limited (Additional File 3).

In our quality assessment, we found that most systematic reviews that fulfilled our inclusion criteria were of high or moderate methodological quality. However, relatively few of the included reviews stated that they had followed the PRISMA or ENTREQ statement, and the compliance with these guidelines can indeed be enhanced – for example, by reporting how many reviewers were involved in the screening of studies and whether they worked independently (PRISMA checklist item 8)[41]. Other shortcomings in the included reviews were inadequate reporting of when in the progression of the disorder the data was collected, and inadequate information on the study authors’ competence in the field. In most reviews, however, a tool for critical appraisal of the original studies had been used, such as the CASP tool.

The major strengths of this meta-review are its broad scope – including three different perspectives of key informants – and the rigorous methodology of the literature screening, which involves systematic assessment of methodological limitations in the included reviews. The tool that we used for assessment of qualitative systematic reviews was developed in parallel to this meta-review and incorporates elements from the PRISMA guidelines [41] and the ENTREQ recommendations [16]. We believe that this tool can also be useful for other authors of qualitative meta-reviews. Another strength of the current study is the adequacy of the data. Most of the findings in our meta-review were based on at least three different systematic reviews and seven to 32 primary studies.

### The study population and research needs

The included reviews focused mainly on anorexia nervosa (AN) or on EDs in general, without specifying a particular diagnosis. None of the identified reviews exclusively evaluated individuals with bulimia nervosa (BN) or binge eating disorder (BED), which was somewhat surprising. The possibility to generalise our findings to other EDs than AN is thus limited. To our knowledge, no systematic review that specifically focuses on experiences of BN or BED have been published after our literature search was performed. Considering the high prevalence of BN and BED that have been reported [6], there is a need to highlight experiences of these disorders in future qualitative systematic reviews.

Most of the included systematic reviews concerned both women and men with EDs, but men were underrepresented relative to their expected incidence, which possibly reflects the proportions of women and men that are studied in the primary qualitative studies of EDs. This is confirmed by a recent systematic review on men’s experiences of ED treatment[42], which identified only nine primary studies (not included in this review since it was published after our literature search). Since the prevalence of EDs is known to be higher among women, there might be a tendency to overlook the problem in men and boys. However, the prevalence of EDs among men has increased substantially over the last two decades and it is estimated that men and boys constitute 20% of all individuals with EDs [7]. Thus, there is an increasing need to acknowledge their experiences, which may not necessarily be interchangeable with those of girls and women.

Since our literature search was performed, a few additional systematic reviews have been published on EDs from the patients’, family members’ and/or health care professionals’ views. These mainly confirm our findings [43–45]. However, there are some interesting new results that build on preexisting knowledge and are worth mentioning. For example one systematic review focused exclusively on the experiences of males with an ED, and besides confirming the findings of this study regarding the call for an individual and person centered approach in treatment, it also added interesting results about recognition, help-seeking and treatment from a male perspective [42].

A systematic review by Johns et al. gave new insights about the perceptions of health care professionals, patients and their family members in terms of meeting professional staff with knowledge about EDs [12]. Another systematic review by Graham et al. described the dilemma faced by health care professionals using the key concept of “coping with caring without curing”, elegantly illustrating the dissonance between careers’ aspiration to help and the reality of their working situation [46].

## Conclusions

To our knowledge, this is the first meta-review of qualitative systematic reviews focusing on experiences of EDs. The compilation acknowledges some important similarities and differences between the views of the three different informants, where health care professionals felt that the biomedical model was helpful, while patients and family members felt that it was insufficient and failed to address their psychological distress. Viewing these perspectives as complementary rather than conflicting may contribute to a better understanding of the complexity of treating EDs. Acknowledging experiences from various perspectives may eventually lead to adaptations in health care that can hopefully improve treatment compliance and recovery rates for individuals with EDs.

Our meta-review also indicates that there is a need for methodologically well conducted qualitative systematic reviews on EDs in which the population is clearly described regarding age, sex, and diagnosis. In particular, there is a need for systematic reviews on experiences regarding BN and BED.

## Supplementary Information

**Additional file 1.** Literature search.

**Additional file 2.** Tool for assessment of methodological limitations

**Additional file 3.** Table of overlapping primary studies

## Data Availability

All relevant data and information are provided in figures, tables and additional files. Additional in-depth information on methods etc. can be provided upon request from the authors.

## Abbreviations

AN: Anorexia nervosa
BED: Binge eating disorder
BN: Bulimia nervosa
CASP: The critical appraisals skills programme
ED: Eating disorders
ENTREQ: Enhancing transparency in reporting the synthesis of qualitative research

## References

[1] Ágh T, Kovács G, Supina D, Pawaskar M, Herman BK, Vokó Z (2016). A systematic review of the health-related quality of life and economic burdens of anorexia nervosa, bulimia nervosa, and binge eating disorder. Eat Weight Disord.

[2] Hay P, Mitchison D, Collado AEL, González-Chica DA, Stocks N, Touyz S (2017). Burden and health-related quality of life of eating disorders, including avoidant/restrictive food intake disorder (ARFID), in the Australian population. J Eat Disord.

[3] van Hoeken D, Hoek HW (2020). Review of the burden of eating disorders: mortality, disability, costs, quality of life, and family burden. Curr Opin Psychiatry.

[4] Samuels KL, Maine MM, Tantillo M (2019). Disordered eating, eating disorders, and body image in midlife and older women. Curr Psychiatry Rep.

[5] Ward ZJ, Rodriguez P, Wright DR, Austin SB, Long MW. Estimation of eating disorders prevalence by age and associations with mortality in a simulated nationally representative US Cohort. JAMA Netw Open. 2019;2(10):e1912925. 10.1001/jamanetworkopen.2019.12925 PMC6802241 31596495

[6] Qian J, Wu Y, Liu F, Zhu Y, Jin H, Zhang H, et al. An update on the prevalence of eating disorders in the general population: a systematic review and meta-analysis. Eat Weight Disord. 2021. 10.1007/s40519-021-01162-z PMC8933366 33834377

[7] Qian J, Hu Q, Wan Y, Li T, Wu M, Ren Z (2013). Prevalence of eating disorders in the general population: a systematic review. Shanghai Arch Psychiatry.

[8] National Institute for Health and Care Excellence. Eating Disorders: Recognition and Treatment. NICE guideline 69. London: National Institute for Health and Care Excellence (UK); 2017.

[9] Hilbert A, Hoek HW, Schmidt R (2017). Evidence-based clinical guidelines for eating disorders: international comparison. Curr Opin Psychiatry.

[10] Staples C, Grunewald W, Smith AR, Rancourt D (2021). Advances in psychotherapy for eating disorders. Adv Psychiat Behav Health.

[11] Steinhausen HC (2009). Outcome of eating disorders. Child Adolesc Psychiatr Clin N Am.

[12] Johns G, Taylor B, John A, Tan J. Current eating disorder healthcare services - the perspectives and experiences of individuals with eating disorders, their families and health professionals: systematic review and thematic synthesis. BJPsych Open. 2019;5(4):e59. 10.1192/bjo.2019.48 PMC6646967 31530301

[13] Lindstedt K, Neander K, Kjellin L, Gustafsson SA (2015). Being me and being us - adolescents' experiences of treatment for eating disorders. J Eat Disord.

[14] Musolino C, Warin M, Wade T, Gilchrist P (2016). Developing shared understandings of recovery and care: a qualitative study of women with eating disorders who resist therapeutic care. J Eat Disord.

[15] Swedish Agency for Health Technology Assessment and Assessment of Social Services (SBU). Eating disorders. An overview of systematic reviews of qualitative research from the perspectives of patients, health care professionals and family members. Stockholm: SBU; 2019. Report No.: 302.

[16] Tong A, Flemming K, McInnes E, Oliver S, Craig J (2012). Enhancing transparency in reporting the synthesis of qualitative research: ENTREQ. BMC Med Res Methodol.

[17] Ouzzani M, Hammady H, Fedorowicz Z, Elmagarmid A (2016). Rayyan-a web and mobile app for systematic reviews. Syst Rev.

[18] Braun V, Clarke V (2006). Using thematic analysis in psychology. Qual Res Psychol.

[19] Fogarty S, Elmir R, Hay P, Schmied V (2018). The experience of women with an eating disorder in the perinatal period: a meta-ethnographic study. BMC Pregnancy Childbirth.

[20] Tierney S, McGlone C, Furber C (2013). What can qualitative studies tell us about the experiences of women who are pregnant that have an eating disorder?. Midwifery.

[21] Thapliyal P, Hay P, Conti J (2018). Role of gender in the treatment experiences of people with an eating disorder: a metasynthesis. J Eat Disord.

[22] Ali K, Farrer L, Fassnacht DB, Gulliver A, Bauer S, Griffiths KM (2017). Perceived barriers and facilitators towards help-seeking for eating disorders: a systematic review. Int J Eat Disord.

[23] Bezance J, Holliday J (2013). Adolescents with anorexia nervosa have their say: a review of qualitative studies on treatment and recovery from anorexia nervosa. Eur Eat Disord Rev.

[24] Eklund R, Salzmann-Erikson M (2016). An integrative review of the literature on how eating disorders among adolescents affect the family as a system—complex structures and relational processes. MENT HEALTH REV J.

[25] Duncan TK, Sebar B, Lee J (2015). Reclamation of power and self: a meta-synthesis exploring the process of recovery from anorexia nervosa. Adv Eat Disord.

[26] Espindola CR, Blay SL (2009). Family perception of anorexia and bulimia: a systematic review. Rev Saude Publica.

[27] Espindola CR, Blay SL (2009). Anorexia nervosa treatment from the patient perspective: a metasynthesis of qualitative studies. Ann Clin Psychiatry.

[28] Espindola CR, Blay SL (2009). Anorexia nervosa's meaning to patients: a qualitative synthesis. Psychopathology.

[29] Fox JR, Dean M, Whittlesea A (2017). The experience of caring for or living with an individual with an eating disorder: a meta-synthesis of qualitative studies. Clin Psychol Psychother.

[30] Medway M, Rhodes P. Young people’s experience of family therapy for anorexia nervosa: A qualitative meta-synthesis. Advances in Eating Disorders. 2016;4(2).

[31] Sibeoni J, Orri M, Colin S, Valentin M, Pradere J, Revah-Levy A (2017). The lived experience of anorexia nervosa in adolescence, comparison of the points of view of adolescents, parents, and professionals: A metasynthesis. Int J Nurs Stud.

[32] Sibeoni J, Orri M, Valentin M, Podlipski MA, Colin S, Pradere J, et al. Metasynthesis of the views about treatment of anorexia nervosa in adolescents: perspectives of adolescents, parents, and professionals. PLoS ONE [Electronic Resource]. 2017;12(1):e0169493. 10.1371/journal.pone.0169493 PMC5215824 28056106

[33] Stockford C, Stenfert Kroese B, Beesley A, Leung N. Women's recovery from anorexia nervosa: a systematic review and meta-synthesis of qualitative research. Brunner-Mazel Eating Disorders Monograph Series. 2018:1–26. 10.1080/10640266.2018.1512301 30247988

[34] de Vos JA, LaMarre A, Radstaak M, Bijkerk CA, Bohlmeijer ET, Westerhof GJ (2017). Identifying fundamental criteria for eating disorder recovery: a systematic review and qualitative meta-analysis. J Eat Disord.

[35] Salzmann-Erikson M, Dahlen J. Nurses' establishment of health promoting relationships: a descriptive synthesis of anorexia nervosa research. J. 2017;26(1):1–13. 10.1007/s10826-016-0534-2 PMC5219017 28111516

[36] McHugh RK, Murray HW, Barlow DH (2009). Balancing fidelity and adaptation in the dissemination of empirically-supported treatments: The promise of transdiagnostic interventions. Behav Res Ther.

[37] Fassino S, Pierò A, Tomba E, Abbate-Daga G (2009). Factors associated with dropout from treatment for eating disorders: a comprehensive literature review. BMC Psychiatry.

[38] Gómez Del Barrio A, Vellisca Gonzalez MY, González Gómez J, Latorre Marín JI, Carral-Fernández L, Orejudo Hernandez S (2019). Characteristics of patients in an eating disorder sample who dropped out: 2-year follow-up. Eat Weight Disord.

[39] Waller G (2016). Treatment protocols for eating disorders: clinicians' attitudes, concerns, adherence and difficulties delivering evidence-based psychological interventions. Curr Psychiatry Rep.

[40] Clinton D (2010). Towards an ecology of eating disorders: creating sustainability through the integration of scientific research and clinical practice. Eur Eat Disord Rev.

[41] Moher D, Liberati A, Tetzlaff J, Altman DG (2009). Preferred reporting items for systematic reviews and meta-analyses: the PRISMA statement. J Clin Epidemiol.

[42] Richardson C, Paslakis G (2021). Men's experiences of eating disorder treatment: A qualitative systematic review of men-only studies. J Psychiatr Ment Health Nurs.

[43] Conti JE, Joyce C, Hay P, Meade T (2020). "Finding my own identity": a qualitative metasynthesis of adult anorexia nervosa treatment experiences. BMC Psychol.

[44] Eaton CM (2020). Eating Disorder Recovery: A Metaethnography. J Am Psychiatr Nurses Assoc.

[45] Wetzler S, Hackmann C, Peryer G, Clayman K, Friedman D, Saffran K (2020). A framework to conceptualize personal recovery from eating disorders: A systematic review and qualitative meta-synthesis of perspectives from individuals with lived experience. Int J Eat Disord.

[46] Graham MR, Tierney S, Chisholm A, Fox JRE (2020). The lived experience of working with people with eating disorders: a meta-ethnography. Int J Eat Disord.

[47] Noblit GWHR (1999). Meta-ethnography: synthesizing qualitative studies.

[48] Thomas J, Harden A (2008). Methods for the thematic synthesis of qualitative research in systematic reviews. BMC Med Res Methodol.

[49] Forsberg C WY. Att göra systematiska litteraturstudier [To conduct systematic literature reviews]. Stockholm: Natur & Kultur. 2013.

[50] Willman A S, P, Bathsevani C. Evidensbaserad omvårdnad: En bro mellan forskning och klinisk verksamhet [Evidence-based nursing: A bridge between research and clinical practice]. Lund: Studentlitteratur. 2006.

[51] Ritchie J SL, O’Connor W. Carrying out qualitative analysis. In: Ritchie J, Lewis J. (Eds.), Qualitative Research Practice: A Guide for Social Science Students and Researchers. Sage, London, Thousand Oaks, New Delhi. 2003.

[52] Whittemore R, Knafl K. The integrative review: updated methodology. J Adv Nurs. 2005;52(5):546–53. 10.1111/j.1365-2648.2005.03621.x 16268861

[53] Evans D. Systematic reviews of interpretive research: interpretive data synthesis of processed data. Aust J Adv Nurs. 2002;20(2):22-6. 12537149

