# Supplementary material for: Experiences of eating disorders from the perspectives of patients, family members and health care professionals: a meta-review of qualitative evidence syntheses
Source: J Eat Disord. 2021 Dec 4;9:156. doi: 10.1186/s40337-021-00507-4 (PMC8642844; doi:10.1186/s40337-021-00507-4)
Supplement: Supplementary file 1 — Additional file 1. Literature search. [file 40337_2021_507_MOESM1_ESM.docx]

# Additional file 1 Literature search

Cinahl via EBSCO 26 September 2018

Subject: eating disorders -systematic reviews of qualitative studies

| Search terms | | Items found |
| --- | --- | --- |
| Population: persons with eating disorders | | |
|  | (MH "Eating Disorders") OR (MH "Anorexia") OR (MH "Anorexia Nervosa") OR (MH "Binge Eating Disorder") OR (MH "Bulimia Nervosa") OR (MH "Bulimia") OR (MH "Night Eating Syndrome") | 10787 |
|  | TI (anorexi* or bulimi* or ((bing* or compulsive*) W2 (eat* or vomit*)) or (eating W2 disorder*) or (food* W2 bing*) or ((self induc* or selfinduc*) W2 vomit*) or (((abnormal* or disturbance* or disturbed or dysfunction* or problem*) W2 eating) or anorectic or (bing* W2 (episode* or meal*)) or EDNOS OR "Purging disorder*" or "Night Eating Syndrome")) OR AB (anorexi* or bulimi* or ((bing* or compulsive*) W2 (eat* or vomit*)) or (eating W2 disorder*) or (food* W2 bing*) or ((self induc* or selfinduc*) W2 vomit*) or (((abnormal* or disturbance* or disturbed or dysfunction* or problem*) W2 eating) or anorectic or (bing* W2 (episode* or meal*)) or EDNOS OR "Purging disorder*" or "Night Eating Syndrome")) | 10432 |
|  | *1 OR 2* | *13175* |
| Study types: meta-synthesis | | |
|  | (ZT "meta synthesis") | 899 |
|  | (MH "Qualitative Studies+") AND ((ZT "meta analysis") or (ZT "systematic review")) | 2338 |
|  | TX (review or systematic or meta or synthes*) N3 (qualitative or narrative or interpret* or integrative or evidence) | 53071 |
|  | TX (meta W3 (study or studies or synthes* or ethnograph* or "data analys*" or summary)) | 11922 |
|  | TX (realist or framework or thematic or "mixed method*") N3 (synthes* or review) | 4599 |
|  | TX ((meta* or review or synthes* or systematic) N3 ("grounded theory")) | 218 |
|  | TX ((information OR data) N2 synthes*) | 7043 |
|  | TX (data W2 extract*) | 14288 |
|  | TX (metasynthes* OR metaethnograph* OR "qualitative cross-case analysis" OR "aggregated analys*" OR "critical interpretive synthesis" or "meta-narrative") | 1027 |
|  | *4 OR 5 OR 6 OR 7 OR 8 OR 9 OR 10 OR 11 OR 12* | *77,541* |
| Study types: systematic reviews | | |
|  | MH "Systematic Review" OR ZT "systematic review" OR MH "Meta Analysis" OR ZT "meta analysis" | 72375 |
|  | (TI (systematic* n3 review*)) or (AB (systematic* n3 review*)) or (TI (systematic* n3 bibliographic*)) or (AB (systematic* n3 bibliographic*)) or (TI (systematic* n3 literature)) or (AB (systematic* n3 literature)) or (TI (comprehensive* n3 literature)) or (AB (comprehensive* n3 literature)) or (TI (comprehensive* n3 bibliographic*)) or (AB (comprehensive* n3 bibliographic*)) or (TI (integrative n3 review)) or (AB (integrative n3 review)) or (JN "Cochrane Database of Systematic Reviews") or (TI (information n2 synthesis)) or (TI (data n2 synthesis)) or (AB (information n2 synthesis)) or (AB (data n2 synthesis)) or (TI (data n2 extract*)) or (AB (data n2 extract*)) or (TI (medline or pubmed or psyclit or cinahl or (psycinfo not "psycinfo database") or "web of science" or scopus or embase)) or (AB (medline or pubmed or psyclit or cinahl or (psycinfo not "psycinfo database") or "web of science" or scopus or embase)) or (TI (meta-analy* or metaanaly*)) or (AB (meta-analy* or metaanaly*)) | 83286 |
|  | *14 OR 15* | *104684* |
| Combined sets & limits (year, language) | | |
|  | *3 AND 13* | *405* |
|  | 17 AND Limiters - Published Date: 19900101-; Language: Danish, English, Norwegian, Swedish | 403 |
|  | *3 AND 16* | *552* |
|  | 19 AND Limiters - Published Date: 19900101-; Language: Danish, English, Norwegian, Swedish | 544 |

The search result, usually found at the end of the documentation, forms the list of abstracts.

AB = Abstract

AU = Author

DE = Term from the thesaurus

MM = Major Concept

TI = Title

TX = All Text. Performs a keyword search of all the  database's searchable fields

ZC = Methodology Index

* = Truncation

“ “ = Citation Marks; searches for an exact phrase

Medline via OvidSP 26 September 2018

Subject: eating disorders -systematic reviews of qualitative studies

| Search terms | | Items found |
| --- | --- | --- |
| Population: persons with eating disorders | | |
|  | "feeding and eating disorders"/ or anorexia nervosa/ or binge-eating disorder/ or bulimia nervosa/ or night eating syndrome/ | 26406 |
|  | (anorexi* or bulimi* or ((bing* or compulsive*) adj2 (eat* or vomit*)) or (eating adj2 disorder*) or (food* adj2 bing*) or ((self induc* or selfinduc*) adj2 vomit*) or (((abnormal* or disturbance* or disturbed or dysfunction* or problem*) adj2 eating) or anorectic or (bing* adj2 (episode* or meal*))) or EDNOS OR "Purging disorder*" or "Night Eating Syndrome").ti,ab. | 50586 |
|  | *1 OR 2* | *56264* |
| Study types: meta-synthesis | | |
|  | exp qualitative research/ AND "review"/ | 2033 |
|  | "mixed methods".ti,ab. AND "review"/ | 837 |
|  | ((review OR systematic OR meta OR synthes*) adj3 (qualitative OR narrative OR interpret* OR integrative OR evidence)).af. | 62359 |
|  | (meta adj3 (study OR studies OR synthes* OR ethnograph* OR "data analys*" OR summary)).af. | 15485 |
|  | ((realist OR framework OR thematic OR "mixed method*") adj3 (synthes* OR review)).af. | 3226 |
|  | ((meta* OR review OR synthes* OR systematic) adj3 "grounded theory").af. | 75 |
|  | ((information OR data) adj2 synthes*).af. | 14808 |
|  | (data adj2 extract*).af. | 47252 |
|  | (metasynthes* OR metaethnograph* OR "qualitative cross-case analysis" OR "aggregated analys*" OR "critical interpretive synthesis" or "meta-narrative").af. | 526 |
|  | *4 OR 5 OR 6 OR 7 OR 8 OR 9 OR 10 OR 11 OR 12* | *127028* |
| Combined sets and limits | | |
|  | *3 AND 13* | *545* |
|  | 14 limited to (yr="1990 -Current" and (danish or english or norwegian or swedish) | 531 |
|  | 3 AND systematic[SB] limited to (yr="1990 -Current" and (danish or english or norwegian or swedish) | 1363 |

The search result, usually found at the end of the documentation, forms the list of abstracts.

.ab. =Abstract

.ab,ti. = Abstract or title

.af.= All fields

Exp= Term from the Medline controlled vocabulary, including terms found below this term in the MeSH hierarchy

.sh.= Term from the Medline controlled vocabulary

.ti. = Title

/ = Term from the Medline controlled vocabulary, but does not include terms found below this term in the MeSH hierarchy

* = Focus (if found in front of a MeSH-term)

* or $= Truncation (if found at the end of a free text term)

.mp=text, heading word, subject area node, title

Multi-database search (ASE, ERIC, Psychology and Behavioral Sciences Collection, SocINDEX) via EBSCO 27 October 2018

Subject: eating disorders -systematic reviews of qualitative studies (narrow, complimentary search)

| Search terms | | Items found |
| --- | --- | --- |
| Population: persons with eating disorders | | |
|  | TI("Eating Disorder" OR "Eating Disorders" OR Anorexi* OR "Binge Eating Disorder" OR Bulimi* OR "Night Eating Syndrome") | 23012 |
| Study types: meta-synthesis | | |
|  | TI (review or systematic or meta or synthes*) N1 (qualitative or narrative or interpret* or integrative or evidence) | 7657 |
|  | TI (meta W1 (study or studies or synthes* or ethnograph* or "data analys*" or summary)) | 1316 |
|  | TI (realist or framework or thematic or "mixed method*") N1 (synthes* or review) | 1064 |
|  | TI ((meta* or review or synthes* or systematic) N1 ("grounded theory")) | 15 |
|  | TI ((information OR data) N1 synthes*) | 295 |
|  | TI (data W1 extract*) | 210 |
|  | TI (metasynthes* OR metaethnograph* OR "qualitative cross-case analysis" OR "aggregated analys*" OR "critical interpretive synthesis" or "meta-narrative") | 332 |
|  | *2 OR 3 OR 4 OR 5 OR 6 OR 7 OR 8 OR 9* | *10390* |
| Combined sets | | |
|  | *1 AND 9* | *29* |
|  | 1 AND 9 Limiters - Published Date: 19900101- | 28 |

The search result, usually found at the end of the documentation, forms the list of abstracts.

AB = Abstract

AU = Author

DE = Term from the thesaurus

MM = Major Concept

TI = Title

TX = All Text. Performs a keyword search of all the  database's searchable fields

ZC = Methodology Index

* = Truncation

“ “ = Citation Marks; searches for an exact phrase

PsycInfo via EBSCO 26 September 2018

Subject: eating disorders -systematic reviews of qualitative studies

| Search terms | | Items found |
| --- | --- | --- |
| Population: persons with eating disorders | | |
|  | DE "Eating Disorders" OR DE "Anorexia Nervosa" OR DE "Binge Eating Disorder" OR DE "Bulimia" OR DE "Purging (Eating Disorders)" | 28613 |
|  | TI (anorexi* or bulimi* or ((bing* or compulsive*) W2 (eat* or vomit*)) or (eating W2 disorder*) or (food* W2 bing*) or ((self induc* or selfinduc*) W2 vomit*) or (((abnormal* or disturbance* or disturbed or dysfunction* or problem*) W2 eating) or anorectic or (bing* W2 (episode* or meal*)) or EDNOS OR "Purging disorder*" or "Night Eating Syndrome")) OR AB (anorexi* or bulimi* or ((bing* or compulsive*) W2 (eat* or vomit*)) or (eating W2 disorder*) or (food* W2 bing*) or ((self induc* or selfinduc*) W2 vomit*) or (((abnormal* or disturbance* or disturbed or dysfunction* or problem*) W2 eating) or anorectic or (bing* W2 (episode* or meal*)) or EDNOS OR "Purging disorder*" or "Night Eating Syndrome")) | 37055 |
|  | *1 OR 2* | *38928* |
| Study types: meta-synthesis | | |
|  | (ZC "metasynthesis") | 238 |
|  | (DE "Qualitative Research" OR DE "Grounded Theory") AND (DE "Literature Review" OR DE "Meta Analysis") | 76 |
|  | TX ((review or systematic or meta or synthes*) N3 (qualitative or narrative or interpret* or integrative OR evidence)) | 28748 |
|  | TX (meta W3 (study or studies or synthes* or ethnograph* or "data analys*" or summary)) | 5508 |
|  | TX ((realist or framework or thematic or "mixed method*") N3 (synthes* or review)) | 2852 |
|  | TX ((meta* or review or synthes* or systematic) N3 ("grounded theory")) | 140 |
|  | TX ((information OR data) N2 synthes*) | 2745 |
|  | TX (data W2 extract*) | 4022 |
|  | TX (metasynthes* OR metaethnograph* OR "qualitative cross-case analysis" OR "aggregated analys*" OR "critical interpretive synthesis" or "meta-narrative") | 640 |
|  | *4 OR 5 OR 6 OR 7 OR 8 OR 9 OR 10 OR 11 OR 12* | *40926* |
| Study types: systematic reviews | | |
|  | DE "Meta Analysis" OR ZC "systematic review" OR ZC "meta analysis" | 37240 |
|  | TX (systematic* N3 review*) OR TX (metaanaly* OR meta-analy* OR "meta analy*") | 56404 |
|  | TX ((systematic* n3 bibliographic*) OR (systematic* n3 literature) OR (comprehensive* n3 literature) OR (comprehensive* n3 bibliographic*) OR (integrative n3 review) OR (information n2 synthesis) OR (data n2 synthesis) OR (data n2 extract*)) OR JN ("Cochrane Database of Systematic Reviews") | 17707 |
|  | *14 OR 15 OR 16* | *64332* |
| Combined sets & limits (language, year) | | |
|  | *3 AND 13* | *523* |
|  | 18 AND Limiters - Publication Year: 1990-2018, Narrow by Language: - english | 499 |
|  | *3 AND 17* | *837* |
|  | 20 AND Limiters - Publication Year: 1990-2018, Narrow by Language: - english | 784 |

The search result, usually found at the end of the documentation, forms the list of abstracts.

AB = Abstract

AU = Author

DE = Term from the thesaurus

MM = Major Concept

TI = Title

TX = All Text. Performs a keyword search of all the  database's searchable fields

ZC = Methodology Index

* = Truncation

“ “ = Citation Marks; searches for an exact phrase

Scopus via Elsevier 26 September 2018

Subject: eating disorders -systematic reviews of qualitative studies

| Search terms | | Items found |
| --- | --- | --- |
| Population: persons with eating disorders | | |
|  | EXACTKEYWORD ("Anorexia Nervosa" OR "Eating Disorder" OR "Eating Disorders" OR "Binge Eating Disorder" OR "Bulimia Nervosa" OR **"**Night Eating Syndrome") | 43298 |
|  | TITLE (anorexi* OR bulimi* OR "eating disorder" OR "eating disorders" OR "binge eating" OR EDNOS OR "purging disorder" or "night eating syndrome") | 30764 |
|  | *1 OR 2* | *50873* |
| Study types: meta-synthesis | | |
|  | EXACTKEYWORD ("meta synthesis") | 539 |
|  | TITLE-ABS-KEY ((review OR systematic OR meta OR synthes*) PRE/3 (qualitative OR narrative OR interpret* OR integrative OR evidence )) | 73902 |
|  | TITLE-ABS-KEY (meta PRE/2 (study OR studies OR synthes* OR ethnograph* OR "data analys*" OR summary )) | 9128 |
|  | TITLE-ABS-KEY ((realist OR framework OR thematic OR "mixed method*" ) PRE/1 ( synthes* OR review)) | 4216 |
|  | TITLE-ABS-KEY (( meta* OR review OR synthes* OR systematic ) PRE/3 ( "grounded theory")) | 98 |
|  | TITLE-ABS-KEY (( information OR data ) PRE/2 synthes*) | 21099 |
|  | TITLE-ABS-KEY (metasynthes* OR metaethnograph* OR "qualitative cross-case analysis" OR "aggregated analys*" OR (data PRE/1 extract*)) | 51706 |
|  | *4 OR 5 OR 6 OR 7 OR 8 OR 9 OR 10* | *144841* |
| Study types: systematic review | | |
|  | EXACTKEYWORD ("Systematic review" ) OR EXACTKEYWORD ("Meta analysis" ) | 265444 |
|  | INDEXTERMS ('systematic review' OR 'meta analysis' OR "Meta-Analysis") | 87492 |
|  | TITLE-ABS-KEY (systematic W/2 review*) OR TITLE-ABS-KEY (meta-analy* OR metaanaly*) OR TITLE-ABS-KEY ("systematic overview*" OR "methodological overview*") | 354336 |
|  | *12 OR 13 OR 14* | *354776* |
| Combined sets | | |
|  | *3 AND 11* | *451* |
|  | **16 AND (( LIMIT-TO ( PUBYEAR , 1990-2018 ) ) AND ( LIMIT-TO ( LANGUAGE , "English " ) OR LIMIT-TO ( LANGUAGE , " swedish " ) OR LIMIT-TO ( LANGUAGE , " danish " ) OR LIMIT-TO ( LANGUAGE , " norwegian " ) )** | **432** |
|  | *3 AND 15* | *1258* |
|  | 18 AND (( LIMIT-TO ( PUBYEAR , 1990-2018 ) ) AND ( LIMIT-TO ( LANGUAGE , "English " ) OR LIMIT-TO ( LANGUAGE , " swedish " ) OR LIMIT-TO ( LANGUAGE , " danish " ) OR LIMIT-TO ( LANGUAGE , " norwegian " ) ) | 1195 |

The search result, usually found at the end of the documentation, forms the list of abstracts.

TITLE-ABS-KEY  = Title or abstract or keywords
**ALL =** All fields
**PRE/n =** "precedes by". The first term in the search must precede the second by a specified number of terms (n).

**W/n =** "within". The terms in the search must be within a specified number of terms (n) in any order.
***** = Truncation

**“ “** = Citation Marks; searches for an exact phrase

LIMIT-TO ( SRCTYPE ,  "j"  = Limit to source type journal
LIMIT-TO ( DOCTYPE ,  "ar"  = Limit to document type article
LIMIT-TO ( DOCTYPE ,  "re"   = Limit to document type review
