# Supplementary material for: Experiences of eating disorders from the perspectives of patients, family members and health care professionals: a meta-review of qualitative evidence syntheses
Source: J Eat Disord. 2021 Dec 4;9:156. doi: 10.1186/s40337-021-00507-4 (PMC8642844; doi:10.1186/s40337-021-00507-4)
Supplement: Supplementary file 2 — Additional file 2. Tool for assessment of methodological limitations [file 40337_2021_507_MOESM2_ESM.pdf]

Tool to assess methodological limitations of qualitative evidence synthesis*

Author(s): ______________________ Year: ____________

Title: ______________________ Reviewed by: ____________

SUMMARY Minor concern ❑ Moderate concern ❑ High concern ❑

INTRODUCTION	YES	NO	NO INFO
1. AIM: Was the research question clearly stated?	❑	❑	❑
2. SEARCH APPROACH: Was the approach to searching for the literature appropriate for the research question?	❑	❑	❑
3. INCLUSION CRITERIA: Were the inclusion/exclusion criteria clearly described?	❑	❑	❑
4. COMPETENCE: Were there a sufficient number of researchers involved in the synthesis who had adequate competence?	❑	❑	❑

COMMENT: ______________________

LITERATURE SEARCH AND SELECTION OF STUDIES	YES	NO	NO INFO
5. SEARCH STRATEGY: Was the search strategy sufficient to capture the relevant literature?	❑	❑	❑
6. STUDY SCREENING: Was the selection of relevant studies conducted independently by more than one reviewer and with consensus?	❑	❑	❑

COMMENT: ______________________

APPRAISAL AND SYNTHESIS OF FINDINGS	YES	NO	NO INFO
7. APPRAISAL: Was risk of bias (or methodological quality) formally assessed using appropriate criteria?	❑	❑	❑
8. APPRAISAL PROCESS: Was the appraisal conducted independently by more than one reviewer and with consensus?	❑	❑	❑
9. SYNTHESIS: Was the synthesis method appropriate for the research question?	❑	❑	❑
10. SYNTHESIS: Was the synthesis conducted appropriately?	❑	❑	❑
11. SYNTHESIS OUTPUT: Were findings clearly grounded in the primary studies?	❑	❑	❑

COMMENT: ______________________

SUMMARIZE THE CONCERNS IDENTIFIED DURING THE ASSESSMENT

Minor concern ❑ Moderate concern ❑ High concern ❑

Reason for concern ______________________

IF APPLICABLE...	YES	NO	NO INFO
A. SYNTHESIS OUTPUT: Did the synthesized result go beyond a summary of results from the included studies?	❑	❑	❑
B. CONFIDENCE IN FINDING: Was the confidence in the findings assessed with GRADE-CERQual in an appropriate way?	❑	❑	❑

* Tong A, Flemming K, McInnes E, Oliver S, Craig J. Enhancing transparency in reporting the synthesis of qualitative research: ENTREQ. BMC Med Res Methodol 2012;12:181.

SBU - Swedish Agency for Health Technology Assessment and Assessment of Social Services
