# Supplementary material for: Experiences of eating disorders from the perspectives of patients, family members and health care professionals: a meta-review of qualitative evidence syntheses
Source: J Eat Disord. 2021 Dec 4;9:156. doi: 10.1186/s40337-021-00507-4 (PMC8642844; doi:10.1186/s40337-021-00507-4)
Supplement: Supplementary file 3 — Additional file 3. Table of overlapping primary studies [file 40337_2021_507_MOESM3_ESM.docx]

# Additional file 3 Overlapping primary studies in the systematic reviews

| Systematic review, Aim and Type of analysis | |
| --- | --- |
| **Fogarty S**, Elmir R, Hay P,Schmied V. The experience of women with an eating disorder in the perinatal period: a meta-ethnographic study. BMC Pregnancy & Childbirth 2018;18:121.  **Aim**  To examine the experience of women with  an eating disorder in the perinatal period:  that is during pregnancy and two years  following birth.  **Type of analysis**  Data analyzed by a meta ethnographic  approach. | **Tierney S**, McGlone C, Furber C. What can qualitative studies tell us about the experiences of women who are pregnant that have an eating disorder? Midwifery 2013;29:542-9.  **Aim**  To synthesise qualitative studies  that focused on the perspective  of women with an ED in relation  to being pregnant.  **Type of analysis**  Data analysis by framework  analysis (Ritchie et al., 2003). |
| Included articles (overlap in blue) | |
| Burton et al. 2015 |  |
| Patel et al. 2005 |  |
| Shaffer et al. 2008 | Shaffer et al. 2008 |
| Stapleton et al. 2008 |  |
| Taborelli et al. 2015 |  |
| Stringer et al. 2010 and  Tierney et al. 2011 | Stringer et al. 2010 and  Tierney et al. 2011 |
| Willis & Rand 1988 |  |
| Hollifield & Hobdy 1990 | Hollifield & Hobdy 1990 |
| Lewis & le Grange 1994 | Lewis & le Grange 1994 |
| Little & Lowkes 2000 |  |
| Namer et al. 1986 |  |
|  | Namir et al. (1986) |
|  | Stapleton (2007) |

| Systematic review, Aim and Type of analysis | | |
| --- | --- | --- |
| de Vos JA, LaMarre A, Radstaak M, Bijkerk CA, Bohlmeijer ET, Westerhof GJ. Identifying fundamental criteria for eating disorder recovery: a systematic review and qualitative meta analysis. Journal of EatingDisorders 2017;5:34.  **Aim**  To identify fundamental criteria for **eating disorder** recovery according to recovered individuals.  **Type of analysis**  Data analysis according to Qualitative meta-analytic approach | Duncan TK, Sebar B, Lee J. Reclamation of power and self: A meta synthesis exploring the process of recovery from anorexia nervosa. Advances in EatingDisorders 2015;3:177-190.  **Aim**  To enhance current understanding  of recovery by synthesising the rich  body of qualitative evidence  examining the phenomenon from  the perspective of those who have  experienced it.  **Type of analysis**  Data analysis a Meta ethnographic  approach | Stockford C, Stenfert Kroese B, Beesley A, Leung N. Women's recovery from anorexia nervosa: a systematic review and meta-synthesis of qualitative research. Brunner-Mazel Eating Disorders Monograph Series 2018:1-26.  **Aim**  To systematically review qualitative studies which have investigated female service users’ experiences of recovering from **AN**.  **Type of analysis**  Data analysed by a Meta  ethnographic approach |
| Included articles (overlap in blue) | | |
| Dawson L et al. 2014 |  | Dawson L et al. 2014 |
| Lamoureux et al. 2005 | Lamoureux et al. 2005 | Lamoureux et al. 2005 |
| Arthur-Cameselle et al. 2014 |  |  |
| Hay PJ et al. 2013 |  | Hay PJ et al. 2013 |
| Lindgren et al. 2015 |  |  |
| Jenkins et al. 2012 | Jenkins et al. 2012 | Jenkins et al. 2012 |
| Linville et al. 2012 |  |  |
| Espindola et al. 2013 | Espindola et al. 2013 | Espindola et al. 2013 |
| Björk et al. 2012 |  |  |
| Nilsson et al. 2006 |  | Nilsson et al. 2006 |
| Bowlby et al. 2012 |  |  |
| Shahar et al. 2012 |  |  |
| Krentz et al. 2005 |  |  |
| Hsu et al. 1992 |  |  |
| Matusek et al. 2009 |  |  |
| Björk et al. 2008 |  |  |
| Pathing et al. 2009 | Pathing et al. 2009 |  |
| Weaver et al. 2005 | Weaver et al. 2005 | Weaver et al. 2005 |
|  | Woods et al. 2004 |  |
|  | Granek et al. 2007 | Granek et al. 2007 |
|  | Federici & Kaplan et al. 2008 | Federici & Kaplan et al. 2008 |
|  |  | Tozzi et al. 2003 |
|  |  | Darcy et al. 2010 |
|  |  | Bradley and Simpson 2014 |
|  |  | Williams et al. 2016 |
|  |  | Smethurst and Kuss 2016 |

| Systematic review, Aim and Type of analysis | | | |
| --- | --- | --- | --- |
| **Espindola** CR, Blay SL. Anorexia nervosa treatment from the patient perspective: a metasynthesis of qualitative studies. Annals of Clinical Psychiatry 2009;21:38-48.  **Aim**  To organize the body of information available in qualitative studies about the treatment of AN.  **Type of analysis**  Data analysed by a meta ethnographic approach | **Medway** M, Rhodes P. Young people’s experience of family therapy for anorexia nervosa: A qualitative meta-synthesis. Advances in Eating Disorders 2016;4.  **Aim**  To describe patient´s experiences of family interventions for AN.  **Type of analysis**  Data analysis based on metasynthesis using thematic synthesis according to Thomas & Harden, 2008. | **Sibeoni** J, Orri M, Valentin M, Podlipski MA, Colin S, Pradere J,et al. Metasynthesis of the Views about Treatment of Anorexia Nervosa in Adolescents: Perspectives of Adolescents, Parents, and Professionals. PLoS ONE [Electronic Resource] 2017;12:e0169493.  **Aim**  To perform a systematic review of qualitative studies to synthesize the views of adolescents with AN, their parents, and their healthcare providers about its treatment.  **Type of analysis**  Data analysed by a Meta ethnographic approach |  |
| Included articles (overlap in blue) | | | |
| Hsu et al. 1992 |  |  |  |
| Surgenor et al (2003) |  |  |  |
| Tozzi et al (2003 |  |  |  |
| Tan et al (2003 |  |  |  |
| Tan et al (2003 |  |  |  |
| Williams et al (2003 |  |  |  |
| Chan and Ma (2003 |  |  |  |
| Colton and Pistrang (2004) |  | Colton and Pistrang (2004) |  |
| Lamoureux and Bottorff (2005 |  |  |  |
| Weaver et al (2005 |  |  |  |
| Redenbach and Lawler (2003) |  |  |  |
| D´Abundo and Chally (2004) |  |  |  |
| Woods (2004 |  |  |  |
| Cockell et al (2004 |  |  |  |
| Keski-Rahkonen and Tozzi (2005 |  |  |  |
|  | Beresin et al. 1989 |  |  |
|  | Chan and Ma (2005) |  |  |
|  | Chen et al. 2010 |  |  |
|  | Goddard et al. (2011) |  |  |
|  | Krautter and Lock (2004) |  |  |
|  | Le Grange and Gelman 1998 |  |  |
|  | Lindstedt et al. (2015) |  |  |
|  | Ma (2008) | Ma (2008) |  |
|  | Ma (2012) |  |  |
|  | Ma and Lai (2007) |  |  |
|  | Maine (1985) |  |  |
|  | Roots et al. (2009) |  |  |
|  | Tantillo et al. (2015) |  |  |
|  | Tierney (2008) | Tierney (2008) |  |
|  | Voriadaki et al. (2015) | Voriadaki et al. (2015) |  |
|  |  | Bakker et al. 2011 |  |
|  |  | Rajman 2004 |  |
|  |  | Rajman and Gill 2012 |  |
|  |  | Beukers 2015 |  |
|  |  | Zugai 2013 |  |
|  |  | Boughtwood and Halse 2009 |  |
|  |  | Boughtwood and Halse 2008 |  |
|  |  | Offord 2006 |  |
|  |  | Tierney 2005 |  |
|  |  | King and Turner 2000 |  |
|  |  | Van Ommen et al. 2009 |  |
|  |  | Freedman 2006 |  |
|  |  | Koruth et al. 2011 |  |
|  |  | Nilsson and Hägglöf 2006 |  |
|  |  | Cottee-Lane et al. 2004 |  |
|  |  | Sharkey-Orgnero 1999 |  |
|  |  | Easter & Tchanturia 2011 |  |
|  |  | McCormarck & McCann 2015 |  |
|  |  | Dallos & Denford 2008 |  |
|  |  | Honey et al. 2007 |  |
|  |  | Bezance & Holliday 2014 |  |
|  |  | Honey & Halse 2007 |  |
|  |  | Engman-Bredvisk et al. 2015 |  |
|  |  | Honey et al. 2006 |  |
|  |  | Jarman 1997 |  |
|  |  | Couturier et al. 2013 |  |
|  |  | Godfrey et al. 2015 |  |
|  |  | Rich 2006 |  |
